# Mobile element warfare via CRISPR and anti-CRISPR in *Pseudomonas aeruginosa*

**DOI:** 10.1093/nar/gkab006

**Published:** 2021-02-05

**Authors:** Lina M León, Allyson E Park, Adair L Borges, Jenny Y Zhang, Joseph Bondy-Denomy

**Affiliations:** Department of Microbiology and Immunology, University of California, San Francisco, San Francisco, CA 94158, USA; Department of Microbiology and Immunology, University of California, San Francisco, San Francisco, CA 94158, USA; Department of Microbiology and Immunology, University of California, San Francisco, San Francisco, CA 94158, USA; Department of Microbiology and Immunology, University of California, San Francisco, San Francisco, CA 94158, USA; Department of Microbiology and Immunology, University of California, San Francisco, San Francisco, CA 94158, USA; Quantitative Biosciences Institute, University of California, San Francisco, San Francisco, CA 94158, USA; Innovative Genomics Institute, Berkeley, CA 94720, USA

## Abstract

Bacteria deploy multiple defenses to prevent mobile genetic element (MGEs) invasion. CRISPR–Cas immune systems use RNA-guided nucleases to target MGEs, which counter with anti-CRISPR (Acr) proteins. Our understanding of the biology and co-evolutionary dynamics of the common Type I-C CRISPR–Cas subtype has lagged because it lacks an in vivo phage-host model system. Here, we show the anti-phage function of a *Pseudomonas aeruginosa* Type I-C CRISPR–Cas system encoded on a conjugative pKLC102 island, and its Acr-mediated inhibition by distinct MGEs. Seven genes with anti-Type I-C function (*acrIC* genes) were identified, many with highly acidic amino acid content, including previously described DNA mimic AcrIF2. Four of the acr genes were broad spectrum, also inhibiting I-E or I-F *P. aeruginosa* CRISPR–Cas subtypes. Dual inhibition comes at a cost, however, as simultaneous expression of Type I-C and I-F systems renders phages expressing the dual inhibitor AcrIF2 more sensitive to targeting. Mutagenesis of numerous acidic residues in AcrIF2 did not impair anti-I-C or anti-I-F function per se but did exacerbate inhibition defects during competition, suggesting that excess negative charge may buffer DNA mimics against competition. Like AcrIF2, five of the Acr proteins block Cascade from binding DNA, while two function downstream, likely preventing Cas3 recruitment or activity. One such inhibitor, AcrIC3, is found in an ‘anti-Cas3’ cluster within conjugative elements, encoded alongside bona fide Cas3 inhibitors AcrIF3 and AcrIE1. Our findings demonstrate an active battle between an MGE-encoded CRISPR–Cas system and its diverse MGE targets.

## INTRODUCTION

The plasticity and rapid evolution of bacterial genomes is driven by the exchange of genetic material between diverse species. This genetic mobility can be blocked by bacterial immune systems, such as restriction enzymes and CRISPR–Cas (Clustered Regularly Interspaced Short Palindromic Repeats and CRISPR associated genes). CRISPR–Cas systems utilize short RNA guides, encoded within a CRISPR array where they are separated by repeat sequences, to direct either a multi-protein (Class 1; Type I, Type III, Type IV) or single protein (Class 2; Type II, Type V or Type VI) effector complex to a matching target on a mobile genetic element (MGE) (1). The targeting paradigm can also be inverted, for example, when the CRISPR–Cas system is encoded by an MGE, such as a lytic bacteriophage, targeting the host (2) or other phages (3).

*Pseudomonas aeruginosa* is an opportunistic human pathogen and also a leading model organism for studies pertaining to bacteriophage-CRISPR interactions and Class 1 CRISPR–Cas biology (4,5). Functional Type I-F (6,7), I-E (8,9) and now IV-A (10) systems have been described, however, a fourth CRISPR–Cas system encoded by this species, the Type I-C system, has not been well characterized (11). Type I-C systems are phylogenetically widespread (12), and can be found in *Streptococcus pyogenes*, *Vibrio*, *Clostridium*, *Neisseria* and *Bacillus* species, but are among the least studied subtypes within the adaptive branch of bacterial immunity. A native *Legionella pneumophila* system was used as a model for spacer acquisition and plasmid targeting (13), while remaining studies of Type I-C systems in *Eggerthella lenta* (14), *Desulfovibrio vulgaris* (15), *Bacillus halodurans* (16) and *Xanthomonas oryzae* (17) have been explored heterologously or *in vitro*, with gaps in our understanding of these systems remaining to be filled. Type I-C systems employ a compact surveillance complex of Cas5, Cas7 and Cas8 with the CRISPR RNA (crRNA) and the *trans*-acting nuclease-helicase, Cas3, which is recruited to cleave and processively degrade DNA (18). These systems lack the common Cas6 crRNA-processing RNase, with Cas5 filling that role (19–21).

CRISPR immunity is often simplified to three stages: adaptation, biogenesis and interference, but a fourth, and equally important facet, is MGE counter-evolution. Anti-CRISPR proteins (Acrs) encoded by MGEs disable CRISPR–Cas systems using diverse mechanisms. Strategies range from blocking DNA binding sites (e.g. AcrIF1, AcrIF2, AcrIF10, AcrIIA2, AcrIIA4) (22–24), to blocking DNA cleavage (e.g. AcrIE1, AcrIF3, AcrIIC1) (9,25,26) and even acting enzymatically to disable CRISPR–Cas (e.g. AcrVA1, AcrVA5, AcrIII-1). Anti-CRISPR discovery efforts continue to yield new biochemical mechanisms for CRISPR–Cas inhibition, while also providing evidence that MGEs encoding *acr* genes face CRISPR–Cas challenge *in situ*. Some Acr proteins are described as ‘broad-spectrum’ due to inhibition of diverged Cas proteins, however, the costs and benefits of this phenotype are yet to be investigated. Here, we describe the MGE targets of the *P. aeruginosa* Type I-C CRISPR–Cas system, which itself is encoded on an MGE, present direct evidence of endogenous Type I-C CRISPR–Cas activity, and report the discovery of seven *Pseudomonas* Type I-C anti-CRISPRs, including four that have dual inhibitory activity.

## MATERIALS AND METHODS

### Microbes

#### Cell culturing

*Pseudomonas aeruginosa* strains (PAO1, PA14 and PA4386) and *Escherichia coli* strains (DH5a) were cultured using lysogeny broth (LB) agar or liquid media at 37 °C supplemented with gentamicin, where applicable, to maintain pHERD30T (50 μg/ml for *P. aeruginos*a, 30 μg/ml for *E. coli*). In all *P. aeruginosa* experiments, expression of genes of interest in pHERD30T was induced using 0.1 % arabinose.

#### Type I-C CRISPR–Cas expression in PAO1

PAO1^IC^ activity was induced using 1 mM IPTG. Construction of this strain is described (27) and may be referred to as LL77 (Targeting crRNA) or LL76 (non-targeting).

#### Bacterial transformations

*P. aeruginosa* transformations were performed using standard electroporation protocols (27). Briefly, overnight cultures were washed twice in an equal volume of 10 % glycerol and the washed pellet was concentrated tenfold in 10% glycerol. These electrocompetent cells were transformed with 20–200 ng plasmid, incubated shaking in LB for 1 h at 37 °C, plated on LB agar with appropriate selection, and incubated overnight at 37 °C. Bacterial transformations for cloning were performed using *E. coli* DH5α (NEB) according to the manufacturer's instructions

#### CRISPRi

CRISPR interference transcriptional repression assays were conducted as in previous work (25). A Δ*cas3* strain was lysogenized with a DMS3m phage encoding an Acr of interest. This lysogen was transformed with a plasmid encoding a crRNA targeting the *phzM* promoter. The crRNA and *cas* genes (in the case of Type I-C) were induced in overnight cultures with 0.25 mM IPTG and 0.05% arabinose. Pyocyanin levels were measured using an acid extraction protocol described previously (25). Pyocyanin quantification was normalized to a strain encoding AcrIIA4, which inhibits Cas9, but not the Type I CRISPR–Cas systems included in this study, resulting in cultures lacking pyocyanin.

### Phages

#### Phage maintenance

*Pseudomonas aeruginosa* DMS3m-like phages (including JBD30 and DMS3m engineered phages) were amplified on PA14 ΔCRISPR, PAO1, or PA4386 *Δcas3* and stored in SM buffer at 4 °C.

#### Construction of recombinant DMS3m acr phages

To generate the isogenic panel of DMS3m and JBD30 anti-CRISPR phages, recombination cassettes were generated with up- and down-stream overhangs to *aca1* and the acr promoter flanking the Acr of interest, as previously described (28). These genes were ordered from TWIST or IDT and were assembled into plasmids using Gibson assembly methods. Recombinant phages were generated by infecting cells transformed with the donor constructs and phages were isolated and assessed for resistance to CRISPR–Cas targeting. The presence of the anti-CRISPR gene was confirmed by PCR. To generate the virulent phages used for liquid growth curve assays, the dms3m c-repressor gene, *gp1*, was mutated using plasmids described previously (28).

#### Plaque forming unit quantification

Phage plaque forming units (PFU) were quantified by mixing 10 μl of phage with 150 μl of an overnight bacterial culture. The infected cells were aliquoted into 3 ml molten 0.7 % top agar and spread on an LB agar plate supplemented with 10 mM MgSO_4_ and appropriate inducers. After 18 hours of growth at 30 or 37 °C, individual plaques were counted. Three biological replicates were done per phage per strain.

#### Phage spot assays

3 ml of molten 0.7 % top agar mixed with 150 μl of bacteria were spread on an LB agar plate supplemented with 10 mM MgSO_4_ to grow a bacterial lawn. Ten-fold serial dilutions of phage were made in SM buffer and 2 μl of each dilution was spotted on the lawn. Plates were incubated at 30 or 37 °C for 16 h and imaged.

#### Efficiency of plaquing (EOP)

EOP was calculated as the ratio of the number of plaque forming units (PFUs) that formed on a targeting strain of bacteria (PAO1^IC^, PA14 WT, PA4386 WT, PaLML1 plus crRNA plasmid) divided by the number of PFUs that formed on a related non-targeting strain (PAO1, PA14 ΔCRISPR, PA4386 ΔCRISPR, PaLML1 plus NT crRNA). Each PFU measurement was performed in biological triplicate. EOP data are displayed as the mean EOP ± standard deviation.

#### Escaper phage isolation

High titer phage preparations were mixed with overnight cultures and spread on an agar plate with top agar. Single plaques that formed after overnight propagation were picked with a sterile pipette tip and resuspended in SM buffer. This process was repeated two times under maintained targeting pressure. The escaper phages were ultimately titered and the protospacer region sequenced.

#### Liquid culture phage infections

PAO1^IC^ was transformed with plasmids encoding either the Type I-C or Type I-F systems from PaLML1 plus one non-DMS3m targeting spacer (‘decoy’ surveillance complexes) to determine the effect of CRISPR–Cas system co-expression. A separate Type I-F plasmid with a Cas8 mutation (K247A) was also constructed. *P. aeruginosa* strains were grown overnight and diluted 100x in LB supplemented with 10 mM MgSO_4,_ gentamicin, 1 mM IPTG, and 0.1 % arabinose. 140 μl of bacterial culture was infected with 10 μl of serially diluted virulent phage in a 96-well plate. Growth and infection was monitored for 20 h using the Synergy H1 microplate reader (BioTek) at shaking at 37°C. Phage was extracted after 20 h by mixing 100 μl of culture from each well with 20 μl chloroform, shaking at RT for 20 min, and centrifugation at 14 000 × g for 2 min.

### Bioinformatics

Numerical data were analyzed in Excel and plotted in GraphPad Prism 6.0.

#### Discovery of acr genes using *aca1* and *aca4*

Anti-CRISPR searches were done as previously described (27).

#### CRISPR array spacer analysis

Spacers were derived from the van Belkum dataset (11) (18 genomes with 12 non-redundant arrays) or from Type I-C containing strains found using BLAST and CRISPRFinder (29) (12 non-redundant arrays). Spacers were analyzed using CRISPRTarget (30) using the Genbank-environmental, RefSeq-plasmid, IMG/VR, and PHAST databases.

PAM analysis was done using the Berkeley Web Logo tool by submitting the upstream and downstream regions flanking the protospacer sequence. These eight nucleotide long flanking sequences are part of the CRISPRTarget output. Every matching protospacer (low cutoff of 20, no redundant matches removed) was utilized for the PAM analysis for *n* = 4443.

To determine the types of elements targeted by the spacers in our collection, the cut-off score was increased to 30 and a PAM match score of +5 was used to narrow the total number of hits to matching elements. If a spacer had multiple matches, the match with the highest score was selected as the representative for that spacer OR the match to a phage genome. Only one match was considered per spacer. This reduced the number of spacers to 131.

Matches were placed into the following categories: Myophages, Siphophages, Podophages, plasmids and assorted prophages. A hit was placed into a phage family, rather than into the prophage category, if the CRISPRTarget output included a link to a specific phage genome. Importantly, this means that being placed into a phage family does not mean that a phage is strictly lytic. Prophages were identified by considering the genes in the protospacer neighborhood.

#### Lineage tracing

For the Type I-C encoding strains from this study, WGS reads were imported from NCBI to Benchling, and the repeats were annotated using the Benchling annotation tool. Individual spacers were extracted using the CRISPRCasFinder tool by copying the entire CRISPR array region. Each spacer sequence was assigned a number, such that identical spacers in distinct strains were assigned the same number, allowing the visualization of spacer similarity across different strains. Lineages were manually curated using the 18 published CRISPR arrays (9) and the additional 12 CRISPR arrays found in this study.

#### Anti-CRISPR phylogenetic tree generation

BLAST was used to generate the tree of AcrIC5 relatives. The following parameters were selected. Tree method: fast minimum evolution. Max seq difference: 0.85. Distance: Grishin (protein).

## RESULTS

### MGE-encoded Type I-C CRISPR–Cas provides immunity in *Pseudomonas aeruginosa*

**Figure 1.**
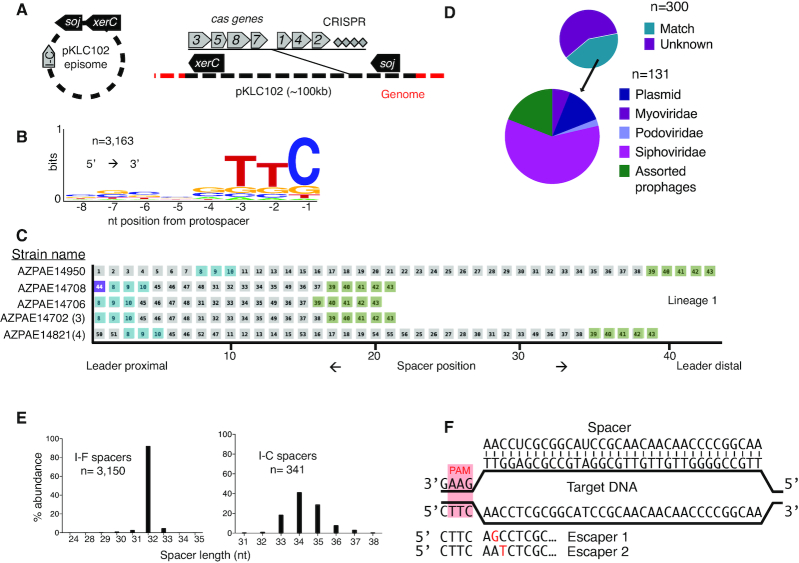
(**A**) *Pseudomonas aeruginosa* Type I-C systems are found on pKLC102 elements. This island can be found integrated into the *P. aeruginosa* genome. Black arrows represent pKLC102 marker genes. *soj* is a chromosome partitioning protein, and *xerC* is a site-specific recombinase. The island can excise to form an episome. (**B**) WebLogo of the region upstream of Type I-C targeted regions showing the consensus PAM to be 5′ TTC 3′. (**C**) CRISPR arrays clustered into lineages based on spacer identity. Lineage 1 is shown here with 5 genomes. Spacer position is marked on the x axis. Spacers that are the same within a lineage are given the same number. Numbers in parentheses following the strain names indicate the number of genomes with the same CRISPR array. The spacer highlighted in purple, #44, is self-targeting. Spacers shared toward the leader proximal region are highlighted in blue. Spacers shared toward the leader distal region are highlighted in green. Shading is meant to facilitate comparisons between related arrays. (**D**) Of the 300 non-redundant spacers, 163 target sequenced genetic elements. Spacers labeled as unknown (dark purple, top pie chart) did not have any matches in sequence databases used by CRISPR Target. Spacers with matches to independent phage genomes (both lytic and temperate) were categorized into three families (siphoviridae, myoviridae, and podoviridae). Spacers that mapped back to phage-like regions in bacterial genomes were categorized as assorted prophages. (**E**) Comparison of spacer lengths found in either Type I-F or Type I-C *P. aeruginosa* CRISPR arrays. (**F**) Schematic of the phage protospacer and CRISPR–Cas spacer. PAM sequence is underlined and highlighted in red. Escaper mutations for phages isolated from PaLML1 are shown below the protospacer sequence with point mutations highlighted in red text.

Type I-C CRISPR–Cas systems previously described in 20 *P*. aeruginosa genomes (11), an environmental isolate in our lab (PaLML1), and 23 additional genomes found using BLAST, are encoded within pKLC102-like elements (Figure 1A). This conjugative element family can be found as either an integrated island or episome in many gram negative bacteria, and is also known as *P. aeruginosa* pathogenicity island (PAPI-1) in some *P. aeruginosa* strains, including PA14 (31–34). It is typically ∼100 kb and while it does not always encode a Type I-C system, it is known to carry virulence factors and increase pathogenicity. To determine if Type I-C CRISPR–Cas is active in *P. aeruginosa*, we first took a bioinformatic approach. While the Cas proteins are highly conserved (90–100% sequence identity) across strains, the CRISPR spacers are diverse (Supplemental Figure S1A and file 1). Alignments of 3163 protospacers with upstream and downstream regions revealed the consensus PAM (Protospacer adjacent motif) to be 5′-TTC-3′, consistent with previous reports (35) (Figure 1B). Among the 42 strains with CRISPR arrays (two published strains have *cas* genes without corresponding arrays), we observed spacer diversity suggestive of active acquisition (Figure 1C and Supplemental Figure S1).

The CRISPR arrays could be clustered into four broad lineages, with strains grouped if they share *at least* one spacer with another array (Figure 1C and Supplemental Figure S1). Some strains have identical CRISPR arrays, which were condensed to one representative per array (see Supplemental File 1 for complete strain information). Strains that cluster together tend to share most of the spacers towards the leader-distal end of the CRISPR array, suggesting that after diverging, each host continues to expand its array independently. For example, strains in lineage 1 share most of their ∼10–15 leader-distal spacers, and then undergo divergence with a series of unique spacers proximal to the leader (Figure 1C). In lineage 2, the diversity is even more striking, as the strains are grouped together by just two ‘core’ spacers (#74 and #75), but have highly distinct arrays, most notably strain AZPAE14395, with ∼40 unique spacers (Supplemental Figure S1). Strains in lineage 3 (PaLML1, AZPAE14876, AZPAE12421, etc.), and lineage 4 (WH-SGI-V-07071, and WH-SGI-V-07073) have completely dissimilar spacers (Supplemental Figure S1), despite having the same frame shift mutation in *cas1* that results in an early stop codon, suggesting continued CRISPR dynamics through an unknown mechanism. In total, there are 300 non-redundant spacers in this collection (Supplemental file 1), and 131 (44%) match sequenced elements, with most spacers targeting phages and prophages (114) and some matching plasmids (17) (Figure 1D). Therefore, although pKLC102 is a ‘selfish’ genetic element, dissection of the Type I-C CRISPR spacer repertoire reveals the immunity module to be ‘domesticated’, targeting canonical bacterial parasites.

In addition to the Type I-C system encoded by strain PaLML1, it also encodes a Type I-F CRISPR–Cas system. The Type I-C spacers cluster with lineage 3, sharing all but one spacer with two of the published CRISPR arrays. We confirmed that the PaLML1 pKLC102 island is also capable of excision, much like the well-studied PAPI-1 of strain PA14 (22) (which lacks a Type I-C CRISPR system), using PCR to amplify the excision junction that forms if the island excises from the chromosome (Supplemental Figure S2A). To verify CRISPR–Cas function, we transformed PaLML1 with a plasmid encoding a Type I-C crRNA targeting phage DMS3m. Because Type I-C spacer length ranges from 32–37 nt, contrary to Type I-F spacers consistently measuring 32 nt (Figure 1E), we tested spacers of each length (i.e. 32 nt, 33 nt, etc.) in PaLML1 to determine their efficacy. Phage targeting occurred in the presence of the phage-specific crRNAs for the I-F system and all crRNA-lengths for the I-C system (Supplemental Figure S2B). Phages that escaped Type I-C targeting were also isolated and Sanger sequencing of the protospacer indicated that these had point mutations at positions +2 and +3 (counting from the PAM) (Figure 1F and Supplemental Figure S2C). This suggests that these nucleotides are part of the seed sequence, a region where mutations are not tolerated for accurate base-pairing with the crRNA. In conclusion, active Type I-C systems in *P. aeruginosa* are on a widespread mobile element, have variable CRISPR spacers suggesting activity *in situ*, and can provide protection against phage.

### Discovery of seven anti-CRISPRs on MGEs that inhibit Type I-C CRISPR–Cas and beyond

Given the diversity of *P. aeruginosa* Type I-C spacers that target MGEs and the robust phage targeting we observed, counter-immunity mechanisms are expected to have manifested due to the threat posed by the Type I-C system. Only one Type I-C anti-CRISPR, AcrIC1, has been previously reported, and it is not found in *P. aeruginosa* (27). To identify additional candidate anti-CRISPR genes, we used previously established bioinformatics approaches: self-targeting (ST) and guilt-by-association (36). Because bacterial genome cleavage is a deadly event (37), a sequenced strain with a CRISPR–Cas system that has a spacer targeting its own chromosome is indicative of some CRISPR inactivation mechanism. Additionally, *acr* genes are often coupled with negative transcriptional regulators known as anti-CRISPR associated (*aca*) genes (38), which can be used to locate candidate *acr* genes (8,27,36). To test candidate Acrs, we used lab strain PAO1 expressing PaLML1’s Cas3–5–8–7 and a phage DMS3m-targeting crRNA from the chromosome, (PAO1^IC^), due to PaLML1’s low transformation efficiency.

**Figure 2.**
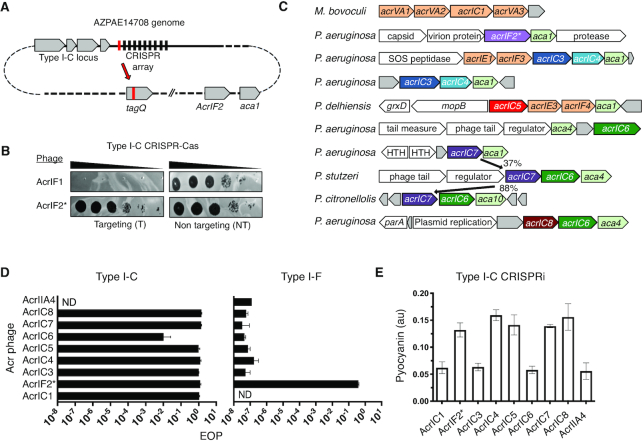
(**A**) Schematic of the self-targeting *P. aeruginosa* strain AZPAE14708 showing the first spacer (in red) targeting *tagQ* and the *aca1* locus encoding acrIF2*. (**B**) A strain expressing the Type I-C CRISPR system in PAO1^IC^ was challenged by phage encoding either AcrIF1 or AcrIF2 in a spot titration plaque assay with ten-fold serial dilutions. (**C**) Gene neighborhood maps of MGEs where new Type I-C acrs (colored, bolded arrows) were identified. Previously discovered acrs (orange), annotated MGE genes (white), and hypothetical genes (gray), are shown. (**D**) Efficiency of plaquing (EOP) calculations for an isogenic panel of phages expressing acrIC genes tested in PAO1^IC^ or PA14 (Type I-F). Each strain was infected in triplicate and plaque counts were averaged and normalized against a strain lacking the indicated CRISPR–Cas system. ND, none detected (**E**) Transcriptional repression via the Type I-C CRISPR system (CRISPRi, strain: PAO1^IC^ Δcas3) and the impact of the acrIC genes. Levels of the pigment pyocyanin are quantified at high levels when CRISPRi is inhibited and low levels when CRISPRi is functional. Each measurement is an average of biological triplicate. A prophage encoding AcrIIA4 was used to lysogenize this same strain as a negative control.

Strain AZPAE14708 encodes a spacer targeting its own type VI secretion gene, *tagQ*, with a perfect protospacer and PAM match (Figure 2A and Supplemental Figure S2D). This spacer is absent in other strains within lineage 1 that share spacer content with AZPAE14708 (Figure 1C). To identify candidate *acr* genes, we used *acr-*associated gene 1 (*aca1*) as an anchor, and found a locus with only the Type I-F inhibitor *acrIF2* (Figure 2A). Surprisingly, expression of AcrIF2 from a phage during infection completely inhibited the Type I-C system (Figure 2B). The dual inhibitory activity was surprising given the evolutionary distance between the I-F and I-C systems (12) (no significant pairwise identity, Supplemental Figure S2E and file 1). Two additional AcrIF2 (hereafter, AcrIF2* to indicate dual specificity) homologues from *Pseudoxanthomonas* and *Stenotrophonomonas*, both associated with *aca1*, with ∼50% sequence identity, were tested and both displayed dual I-C and I-F activity (Supplemental Figure S2F). Strains from these genera also encode Type I-C and Type I-F systems.

acrIF2* is very narrowly distributed and thus we reasoned that more Type I-C Acrs likely exist. Using *aca1* and *aca4* as marker genes, 27 *aca*-associated candidates were tested (Supplementary Table S2), revealing six more Type I-C inhibitors in a series of distinct MGEs including plasmids, transposons, conjugative elements, and phages (Figure 2C and Supplementary Table S1). Many of the MGEs frequently targeted by pKLC102-encoded Type I-C spacers harbor one or more of these seven new inhibitors (Supplemental Figure S3A). An additional gene was identified that solely inhibited the *P. aeruginosa* Type I-E system, *acrIE9* (discussed below). This collection consisted of genes associated with *aca1* (*acrIC3*, *acrIC4* and *acrIC5*) or *aca4* (*acrIC6*, *acrIC7* and *acrIC8*). Each of the new *acr* genes were identified in *P. aeruginosa*, except *acrIC7*, which was first identified in *P. stutzeri* (*acrIC7_Pst_*) adjacent to *aca4* (Supplemental Figure S3B). A homologue was found in *P. citronellolis* (*acrIC7_Pci_*, 88% amino acid sequence identity), adjacent to a new helix-turn-helix transcriptional regulator, which we have named *aca10*. In both instances, *acrIC6* is also present in the locus. An *aca1*-adjacent distant *acrIC7* homologue was also found in *P. aeruginosa* (37% sequence identity), although it did not confer Type I-C anti-CRISPR activity (Supplemental Figure S3B and C).

A panel of isogenic DMS3m phages was engineered to express each individual *acr* gene, including a negative control (Cas9 anti-CRISPR, *acrIIA4*). Efficiency of plaquing (EOP) was assessed during infection of PAO1^IC^ (Figure 2D). Each phage had an EOP ≈ 1 when infecting cells expressing the Type I-C system, except AcrIC6, which appeared to be quite weak (EOP ≈ 0.01). Only AcrIF2* had activity against the Type I-F system, with an EOP ≈ 1, compared to EOP ≈ 10^−7^ for all other Acr proteins.

To determine how the new Acrs inhibit the Cas machinery, we tested whether they alleviate CRISPR transcriptional interference (CRISPRi) in a Δ*cas3* background, a readout for inhibited DNA-binding by Cascade. A colorimetric assay was adapted from previous work (7), using a Type I-C crRNA to repress transcription of the *phzM* gene. If CRISPRi is functional, the surveillance complex blocks *phzM* transcription, turning the *P. aeruginosa* culture yellow. If DNA-binding is inhibited, the culture is a natural blue-green (Supplemental Figure S3D). Five of the proteins, AcrIF2*, IC4, IC5, IC7_Pst_ and IC8, blocked CRISPRi. Expression of AcrIC1 (a previously discovered protein from *Moraxella* (27)) and AcrIC3, however, did not interfere with CRISPRi, suggesting that they bind to Cas3 or Cascade in a way that prevents Cas3 recruitment or DNA cleavage, while allowing Cascade-DNA binding (Figure 2E, Supplementary Table S1). AcrIC6 did not block CRISPRi but given its weak activity, we are hesitant to interpret this negative result.

### Multi-system inactivation by AcrIF2*

**Figure 3.**
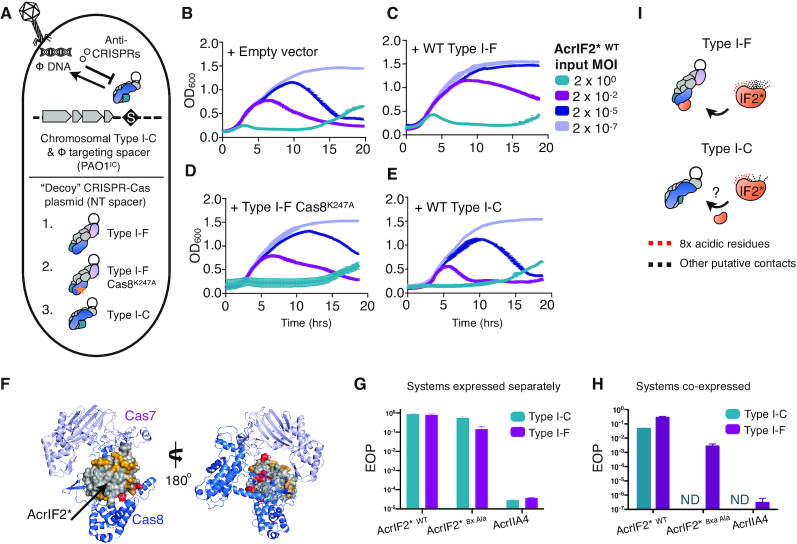
(**A**) Schematic of the experiment performed in panels 3b-3e. The PAO1^IC^ strain, with the I-C system and phage-specific crRNA integrated into the chromosome was transformed with plasmids encoding ‘decoy’ CRISPR–Cas surveillance complexes, which contain a non- DMS3m targeting crRNA. A Type I-F Cas8^K247A^ mutant, which loses affinity for the AcrIF2 inhibitor was included as a control. (**B**–**E**) Liquid infection assay with PAO1^IC^ transformed with indicated ‘decoy’ surveillance complex plasmids and infected with a virulent DMS3m phage expressing AcrIF2* ^WT^. Strains were grown in a plate reader over 20 h with OD_600_ tracked over time. (**F**) Color-coded structure of AcrIF2* bound to the Type I-F surveillance complex (PDB: 5UZ9). The Type I-F surveillance complex is shown as a ribbon with Cas8 (blue), and one Cas7 monomer (lilac), AcrIF2* (grey space-filling model), and mutated amino acids (red) and remaining acidic residues (yellow) shown. (**G**) Quantification of the efficiency of plaquing (EOP) on PAO1^IC^ or PA14 for phages expressing the indicated acr gene. (**H**) Quantification of the efficiency of plaquing (EOP) on PaLML1 for phages expressing the indicated acr gene. (**I**) Schematic representation of AcrIF2 binding to the Type I-F or Type I-C surveillance complex. Red lines are representative of the eight mutated acidic residues. Black lines represent other non-covalent contacts. Because AcrIF2* has stronger activity against the Type I-F system, more black lines are used to represent its additional contacts that mediate activity against Type I-F versus Type I-C.

Most AcrIC proteins identified here are acidic proteins that block DNA-binding (Supplementary Table S1), thus we focused on the well-studied AcrIF2* as a model protein for ecological and mechanistic experiments. AcrIF2* directly prevents the Type I-F CRISPR surveillance complex from binding to DNA (23,24,25). While many MGEs encode distinct inhibitors of Type I-C, I-E and I-F systems, AcrIF2* can be found encoded alone and thus we wondered whether a phage expressing this protein can inhibit both systems simultaneously. It has previously been reported that a phage concentration threshold, inversely proportional to Acr strength, is needed to inhibit CRISPR–Cas targeting (28,39). To measure the strength of this bi-functional Acr protein, we infected cells expressing the Type I-C system and a targeting spacer (PAO1^IC^) from the chromosome, plus a variable ‘decoy’ complex with a non-targeting spacer from a plasmid, with our Acr phages (Figure 3A). The AcrIF2*-expressing phage causes collapse of the culture at low infectious doses (Figure 3B), similar to AcrIC1, an anti-CRISPR that only inhibits Type I-C (Supplementary Figure S5D–F). However, when the Type I-F system with a non-targeting crRNA was co-expressed in this strain, the AcrIF2* encoding phage required an initial MOI of 2 × 10^−2^ to lyse the culture, compared to 2 × 10^−5^ with empty vector (Figure 3C), while the AcrIC1 phage was unaffected (Supplementary Figure S5D). Interestingly, when cells expressed the Type I-F system with a Cas8 mutation (K247A) which was previously shown to reduce AcrIF2* binding (23), an intermediate AcrIF2* phage concentration was sufficient for culture collapse (Figure 3D). Phage output from these experiments was also quantified. In the presence of the decoy Type I-F system, phage output at the two lowest MOIs was reduced by ∼100–1000-fold compared to decoy Type I-F Cas8^K247A^, Type I-C, or the non-targeting control (Supplemental Figure S6A–D). As a control, over-expression of more Type I-C complexes had little impact on concentration thresholds (Figure 3E and Supplemental Figure S6C), and a phage encoding AcrIIA4, a Cas9 inhibitor, was ineffective at causing culture lysis, except at the highest MOI under all conditions tested (Supplementary Figure S5G–I). Therefore, it appears that during phage infection there is a cost to being a dual inhibitor, where the ability of AcrIF2* to inhibit the Type I-C system is weakened by the presence of the Type I-F system, while monotypic AcrIC1 retained its potency.

AcrIF2* inhibits the Type I-F complex by interacting with key PAM-binding residues on Cas7 and Cas8, as revealed by previous cryo-EM and crystallography studies (Figure 3F). We therefore next sought to determine whether AcrIF2* acidic residues in this interface are required for inhibition or impact the observed competition defect. Of AcrIF2*’s 96 residues, 24% are acidic, giving it an overall negative charge (p*I* = 4.0). Despite Cas proteins from Type I-C and I-F having completely distinct sequences (Supplemental Figure S2E), this negative surface charge could allow AcrIF2* to block both the I-C and I-F DNA recognition motifs. We selected eight AcrIF2* residues (D30, E36, D76, E77, E82, E85, E91, E94) that sit within ∼5 Å of a basic residue on Type I-F Cas7/Cas8 (Figure 3F and Supplemental Figure S4A) and incrementally mutagenized them. All of the plasmid-expressed mutants, up to an 8× Ala mutant (acrIF2*^8xAla^) surprisingly maintained Acr activity against the Type I-F and I-C systems, while more dramatic mutations (e.g. the eight selected residues mutated to lysine or the 8 residues mutated to glutamine or asparagine) lost all inhibitory function (Supplemental Figure S4B). When the 8× Ala mutant was expressed from the endogenous phage *acr* locus, it also retained function against the Type I-C and Type I-F systems individually (Figure 3G), which was surprising given the presumed reliance on these negatively charged residues. However the 8x Ala mutant phage was inactivate against the Type I-C system (EOP < 10^−4^) when infecting a strain expressing both Type I-F and I-C (PaLML1), again consistent with a dual inhibition cost (Figure 3H). Activity against the I-F system was only partially weakened (EOP = 0.02, Figure 3G, H, and Supplemental Figure S4C), however. The more sensitive phage concentration threshold infection assay that revealed the competition cost for WT AcrIF2* also indicated that the mutant had a very weak anti-CRISPR phenotype, only providing protection to the phage at the highest MOI when faced with the targeting I-C and decoy I-F complexes (Supplemental Figure S5B and C). Output PFUs were again assessed, revealing that mutant AcrIF2* phage output was reduced ∼100 000-fold when competing with WT Type I-F or Type I-F Cas8^K247A^ decoy systems, but less so with excess Type I-C, compared to the NT control (Supplemental Figure S6E–H). Interestingly, this reduction in mutant AcrIF2* output phage was the same for WT and Type I-F Cas8^K247A^ decoy systems (Compare Supplemental Figure S6E and 6F), suggesting that mutant AcrIF2* retains similar binding affinity for WT Cas8 versus Cas8^K247A^. Overall, AcrIF2* strength is completely context-dependent, exhibiting a conditional defect in the presence of two competing surveillance complex binding targets. This defect, however, is minimized by the excessive negativity of the protein, as many of these residues are not required for function, per se, but help to buffer the defect generated by *in vivo* competition. The weakened activity of the mutated AcrIF2* protein against the Type I-C system is consistent with non-identical, but perhaps overlapping, AcrIF2* binding interfaces. We posit that WT AcrIF2* may make more contacts with the Type I-F surveillance complex vs. the Type I-C surveillance complex, stabilizing its interaction with the former (Figure 3I).

### Broad-spectrum inhibitory activity by the I-C anti-CRISPRs

**Figure 4.**
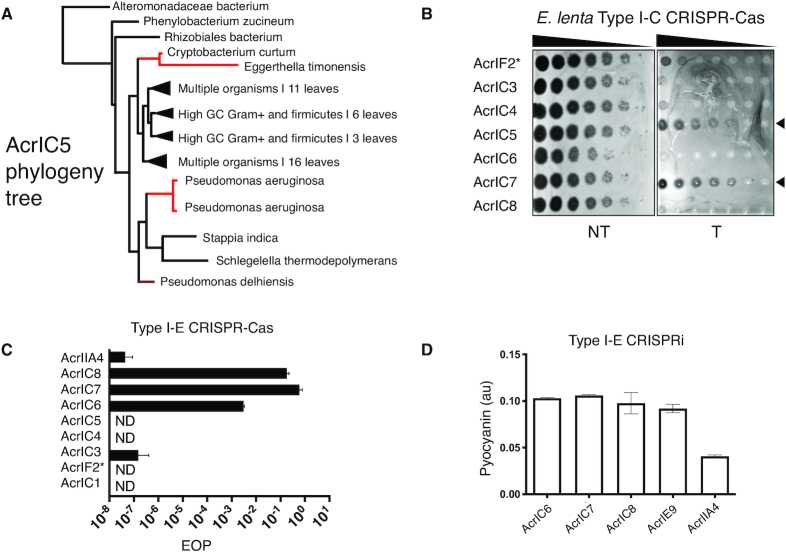
(**A**) Phylogenetic tree of AcrIC5 protein showing its broad distribution. Lines highlighted in red denote strains of relevance in this work. Tree was generated using BLAST pairwise alignments (**B**) Plaque assay of acr-encoding engineered JBD30 phages tested against the *E. lenta* Type I-C system expressed heterologously in *P. aeruginosa*. Phage was serially diluted 10× for each spot. (**C**) EOP calculations for an isogenic panel of phages encoding the indicated acr gene, infecting a strain expressing the Type I-E CRISPR–Cas system (PA4386). Each bar is the average of infections done in biological triplicate normalized to the number of plaques on PA4386 Δcas3. (**D**) Type I-E CRISPRi, conducted as in Figure 3 (host: PA4386 Δcas3) with the Acr proteins that inhibit Type I-E function assayed. AcrIIA4 is a negative control.

We next surveyed the phylogenetic distribution of the new *acr* genes reported here. Most of the Acr proteins were limited to a single genus: AcrIC1 to *Moraxella*, and AcrIF2*, AcrIC3, AcrIC4 and AcrIC7 were only found in *Pseudomonas*. AcrIC5 orthologues were found distributed across Proteobacteria, Firmicutes, and Actinobacteria (Figure 4A), and AcrIC8 orthologues were found sparingly in *Pseudomonas*, Spirochetes, and Rhizobiales. AcrIC6 can be found broadly in various classes (Alpha-, Beta- and Gamma-proteobacteria) with many homologues in *Salmonella enterica*. We took note of Actinobacterial AcrIC5 homologues in the human-associated microbes *Cryptobacterium curtum* and *Eggerthella timonensis*, given that an active *Eggerthella lenta* Type I-C CRISPR–Cas system was described recently (14). We tested whether a phage encoding the *Pseudomonas* AcrIC5 homologue could inhibit the *E. lenta* I-C system heterologously expressed in *P. aeruginosa* and observed strong anti-CRISPR function (Figure 4B), despite *cas* gene sequence identities between 35 and 55% (Supplemental Figure S7A). Surprisingly, AcrIC7 also inhibited the *E. lenta* I-C system, despite no identified homologues outside of the *Pseudomonas* genus.

The broad-spectrum activity of AcrIF2* (I-F and I-C), AcrIC5 (I-C*_Pae_* and I-C*_Ele_*), and AcrIC7 (I-C*_Pae_* and I-C*_Ele_*), motivated us to test the new Acr proteins against another system found in *P. aeruginosa*, Type I-E. Type I-C, Type I-F and Type I-E systems are phylogenetically distinct subtypes, with I-F and I-E systems sharing a more recent common ancestor. AcrIC7*_Pst_, AcrIC7*_Pci_, AcrIC7*_Pae_ and AcrIC8*, inhibited the Type I-E system well, while AcrIC6* was again, a weak anti-CRISPR (Figure 4C, AcrIC8 locus map in Supplemental Figure S7B). The new Type I-E Acr proteins (AcrIC6*, AcrIC7*_Pst_, AcrIC8* and AcrIE9) all inhibited Type I-E CRISPRi (Figure 4D), indicating that they block DNA binding. Curiously, AcrIC7_Pae_*only* inhibited the I-E subtype, unlike its dual I-C/I-E inhibiting homologues (Supplemental Figures S3C and S7C). Searching through sequenced genomes revealed that *P. stutzeri* and *P. aeruginosa* encode both I-C and I-E subtypes, while *P. citronellolis* encodes only Type I-F systems.

### Anti-CRISPRs that inhibit DNA cleavage by Cas3

**Figure 5.**
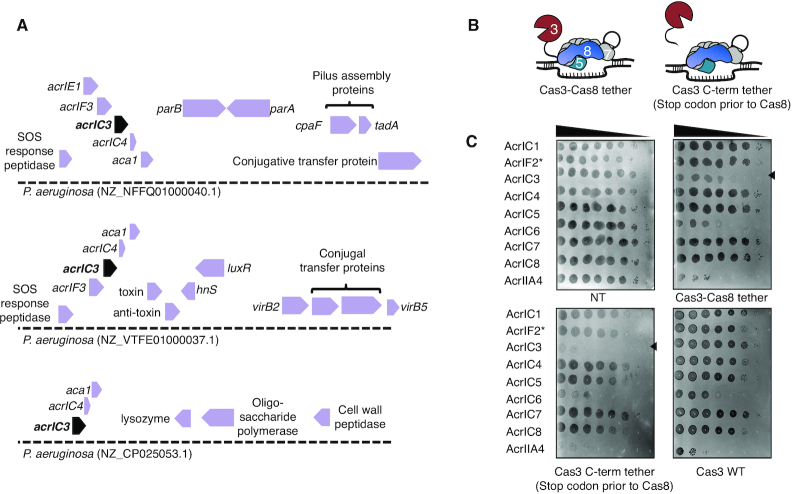
(**A**) Gene loci showing *acrIC3*. *acrIC3* is found on various MGEs, and is often associated with AcrIE1 and AcrIF3, which are Cas3 interacting proteins. (**B**) Schematic of the Type I-C mutant where the C-terminus of Cas3 is tethered to the N-terminus of Cas8 with a short linker peptide (‘Cas3-Cas8 tether’) and a related construct where a stop codon is added after the linker peptide (‘Cas3 C-term tether’). (**C**) Spot titration plaque assay with ten-fold serial dilutions of phage showing the plaquing efficiency of Acr-expressing DMS3m phages on non-targeting (NT), or Type I-C expressing strains, either with Cas3–Cas8 tethered, Cas3 C-term tether, or Cas3 WT.

Acr proteins that allow for DNA binding but still block phage DNA cleavage, like AcrIC1 and AcrIC3 (Figure 2E), effectively turn the endogenous CRISPR–Cas machinery into a catalytically dead, transcriptional repression system. *acrIC3* can be frequently found flanked by *acrIE1* and *acrIF3* in *P. aeruginosa*, two Cas3 inhibitors that enable CRISPRi (9,25). This reveals a remarkable ‘anti-Cas3 locus’ for all three Type I CRISPR systems in *P. aeruginosa* (Figure 5A). Conjugative transfer, *parA/B* genes, and type IV secretion system genes are found flanking these *acr* genes. The role that an ‘anti-Cas3 island’ may play in conjugative transfer from cell to cell is yet to be determined, but this phenomenon may indicate that neutralizing the ssDNAse Cas3 is an effective means to ensure successful transfer, which proceeds through a ssDNA intermediate. When not found with other CRISPRi-enabling inhibitors, *acrIC3* is carried by phages, along with *acrIC4*, which is always paired with *acrIC3*. *acrIC1* is found on *Moraxella* phages and prophages, flanked by *acrVA1, acrVA2* and *acrVA3*, genes encoding Cas12 inhibitor proteins. 

In an effort to distinguish the inhibitory mechanisms for AcrIC1 and AcrIC3, we constructed a Type I-C complex where the Cas3 C-terminus was tethered to the Cas8 N-terminus with a 13 amino acid sequence (RSTNRAKGLEAVS) (Figure 5B). This construct was inspired by, and designed to mimic, naturally occurring variants of Type I-E systems in *Streptomyces griseus*, which encode Cas3 and Cas8 as a single polypeptide, with the same short linker peptide in between (40). A control strain with a stop codon immediately following the C-terminal Cas3 tag was also constructed (Figure 5B). A similar fusion of Cas3 to Cas5, which is seen in some Type I-C systems, was inactive when tested (not shown). When the panel of Type I-C Acr-expressing phages infected a strain expressing this minimal system, the fusion efficiently evaded the AcrIC3 protein, targeting this phage by ∼1,000-fold, while all other *acr* phages, with the exception of AcrIC6, replicated well (Figure 5C). A version of Cas3 just possessing the linker on its C-terminus surprisingly also blocked the activity of AcrIC3, suggesting that AcrIC3 directly interacts with the C-terminus of Cas3, but the linker residues block this interaction. This demonstrates that AcrIC1 and AcrIC3 utilize distinct mechanisms to inhibit the Type I-C system downstream of DNA-binding.

## DISCUSSION

Organisms encoding CRISPR–Cas immune systems are locked in battle with genetic parasites that encode anti-CRISPR proteins capable of disabling CRISPR–Cas activity (4). However, the Type I-C system in *P. aeruginosa* is also found on a common MGE (pKLC102) that can exist as either an island or as a plasmid (33,34). Since mobile elements (here, encoding CRISPR–Cas or anti-CRISPRs) can transfer antibiotic resistance genes, virulence factors, immune systems, and other fitness-altering genetic material to their host (41,42), this generates an interesting paradigm for CRISPR and anti-CRISPR interactions (43). Mobile CRISPR–Cas systems can deliver immunity horizontally, transferring not only *cas* genes, but also a library of spacers against *other* MGEs. As CRISPR–Cas systems have been identified on plasmids (44) and phages (2,3), this phenomenon could be highly prevalent.

**Figure 6.**
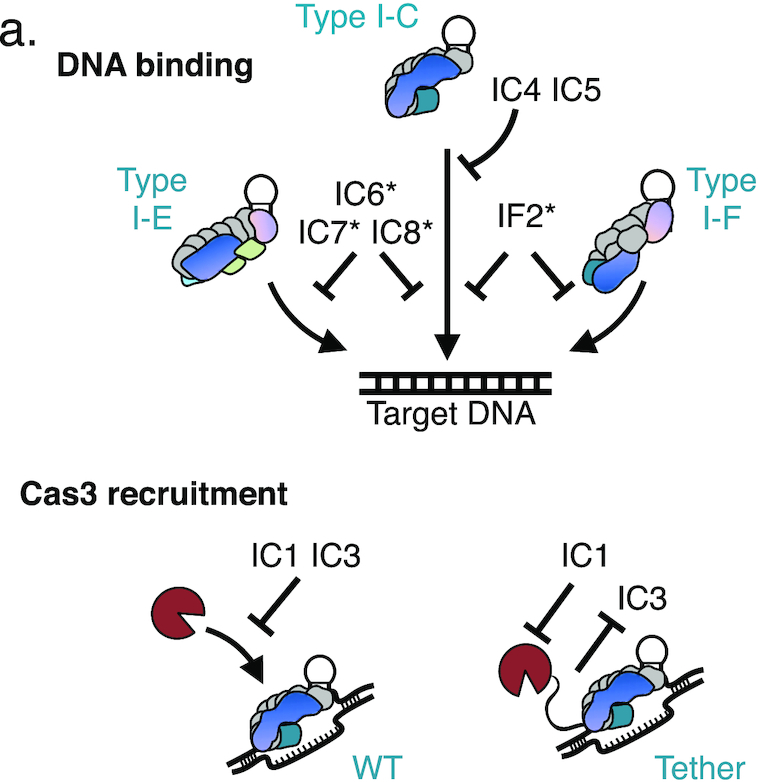
(**A**) Schematic representation of the three Type I subtypes investigated and the novel cognate anti-CRISPRs that inhibit them.

The role of Acr proteins in the dissemination and maintenance of MGEs in bacterial genomes is just beginning to be explored (45). The Acrs described in this study were found encoded by diverse MGEs that are frequent targets of the *P. aeruginosa* Type I-C spacer repertoire (Supplemental Figure S3A). AcrIC1, AcrIF2*, AcrIC5, AcrIC6* and AcrIC7* are commonly found within phages, while AcrIC6* and AcrIC8* are associated with Tn3 family transposases (Supplemental Figure S7E and S7F). Acr proteins facilitate the maintenance of prophages in genomes encoding spacers against that phage, which can help maintain CRISPR–Cas by preventing self-targeting, and even weak Acr proteins can overcome kinetic limitations by working cooperatively (27,28,36,39,46). Additionally, multi-system inhibition may be commonly exploited by MGEs, since bacteria are not limited to only one CRISPR–Cas subtype (See summary of our data in Figure 6A). Such a tactic conserves genetic real estate, and acts as insurance against the threat of assorted immune systems, but may have a negative impact on fitness, as we demonstrated with AcrIF2*.

Of the eight Type I-C anti-CRISPR proteins, all but one (AcrIC8*) had high acidic amino acid content. (Supplementary Table S1). This has been a common theme among Acr proteins and inhibitors of other immune systems (47). Excess acidic residues could help stabilize binding to diverse Cas proteins, provide essential residues for inhibiting more than one system, and buffer against *in vivo* competition with multiple binding partners. For example, the T7 phage encoded Ocr protein is highly acidic and forms a dimer with a bend similar to B-DNA (48,49). Ocr was initially discovered as a Type I restriction enzyme system inhibitor and was more recently shown to inhibit the anti-phage system, BREX (50). Importantly, systematic mutation of Ocr's acidic residues revealed it to be highly recalcitrant to breakage, similar to AcrIF2*, maintaining inhibitory activity against Type I R-M even with up to 33% of acidic residues mutated (48). Similarly, Cas9 inhibitors AcrIIA2 and AcrIIA4 are highly acidic, can inhibit diverged Cas9 orthologues (36), and have been subjected to extensive mutagenesis, also appearing to have dispensable acidic residues (22). AcrIF2* can also be considered a DNA mimic or a DNA competitor, with structural work showing that it partially overlaps with the PAM binding site (23,24,51), and our mutagenesis demonstrating that it is also quite resilient. This suggests that DNA mimicry is a potent and flexible anti-immune strategy.

Our work here underscores the importance of studying CRISPR–Cas and Acr mechanisms *in vivo*, revealing multiple new insights, including broadly inactivating anti-CRISPR proteins encoded by various MGEs and the flexibility of DNA mimicry, a common anti-CRISPR and anti-immune strategy. We propose that these DNA mimics are excessively negative to broaden their inactivation potential and buffer against competition and co-evolution in the DNA-binding pocket for CRISPR–Cas systems. Together with the spacer diversity uncovered, functional phage interference demonstrated, and the discovery of numerous diverse anti-CRISPR proteins encoded by *P. aeruginosa* mobile genetic elements, we conclude that the mobile Type I-C CRISPR–Cas system in *P. aeruginosa* is functional in nature. These observations further bolster our understanding of the importance of CRISPR–Cas to the biology of this species and generate a model organism for future Type I-C CRISPR–Cas work.

## DATA AVAILABILITY

Anti-CRISPR and Aca protein NCBI protein accession codes are as follows: AcrIC1 (WP_046701304.1), AcrIF2 (WP_015972868.1), AcrIC3 (WP_058130594.1), AcrIC4 (WP_153575361.1), AcrIC5 (WP_089394111.1), AcrIC6 (WP_080050315.1), AcrIC7 (WP_003294373.1), AcrIC8 (WP_074202337.1), Aca10 (WP_074980464.1), AcrIE9 (WP_101192668.1). Sequences can be accessed at https://www.ncbi.nlm.nih.gov.

## Supplementary Material

gkab006_Supplemental_Files
