# Supplementary material for: Mobile element warfare via CRISPR and anti-CRISPR in Pseudomonas aeruginosa
Source: Nucleic Acids Res. 2021 Feb 5;49(4):2114–25. doi: 10.1093/nar/gkab006 (PMC7913775; doi:10.1093/nar/gkab006)
Supplement: gkab006_Supplemental_Files [file gkab006_supplemental_files.zip › Supplementary figures Dec072020.pdf]

Supplementary table 2

Candidate Number	Accession	Anti-CRISPR identity	*aca* association
1	KSR23770.1	AcrIC3	*aca1*
2	KSO29066.1	N/A	*aca1*
3	KSL61975.1	N/A	*aca1*
4	SDK41378.1	AcrIC5	*aca1*
5	CDO85538.1	AcrIC4	*aca1*
6	WP_085056855.1	N/A	*aca1*
7	WP_047296680.1	N/A	*aca1*
8	WP_092238848.1	N/A	*aca1*
9	WP_044274829.1	N/A	*aca1*
10	WP_071574229.1	N/A	*aca1*
11	WP_023657539.1	N/A	*aca1*
12	ABR13386.1	N/A	*aca4*
13	ABR13387.1	N/A	*aca4*
14	SDJ61905.1	N/A	*aca4*
15	OPE29935.1	N/A	*aca4*
16	OPD90261.1	N/A	*aca4*
17	WP_060613673.1	N/A	*aca4*
18	WP_080050315.1	AcrIC6*	*aca4*
19	EWC40192.1	AcrIC7*	*aca4*
20	GCA55691.1	N/A	*aca4*
21	WP_101192668.1	AcrIE9	*aca4*
22	WP_101192667.1	N/A	*aca4*
23	WP_101192666.1	N/A	*aca4*
24	WP_045884682.1	N/A	*aca4*
25	WP_045884679.1	N/A	*aca4*
26	WP_074202337.1	AcrIC8*	*aca4*
27	WP_074202338.1	N/A	*aca4*

Supplemental figure 1

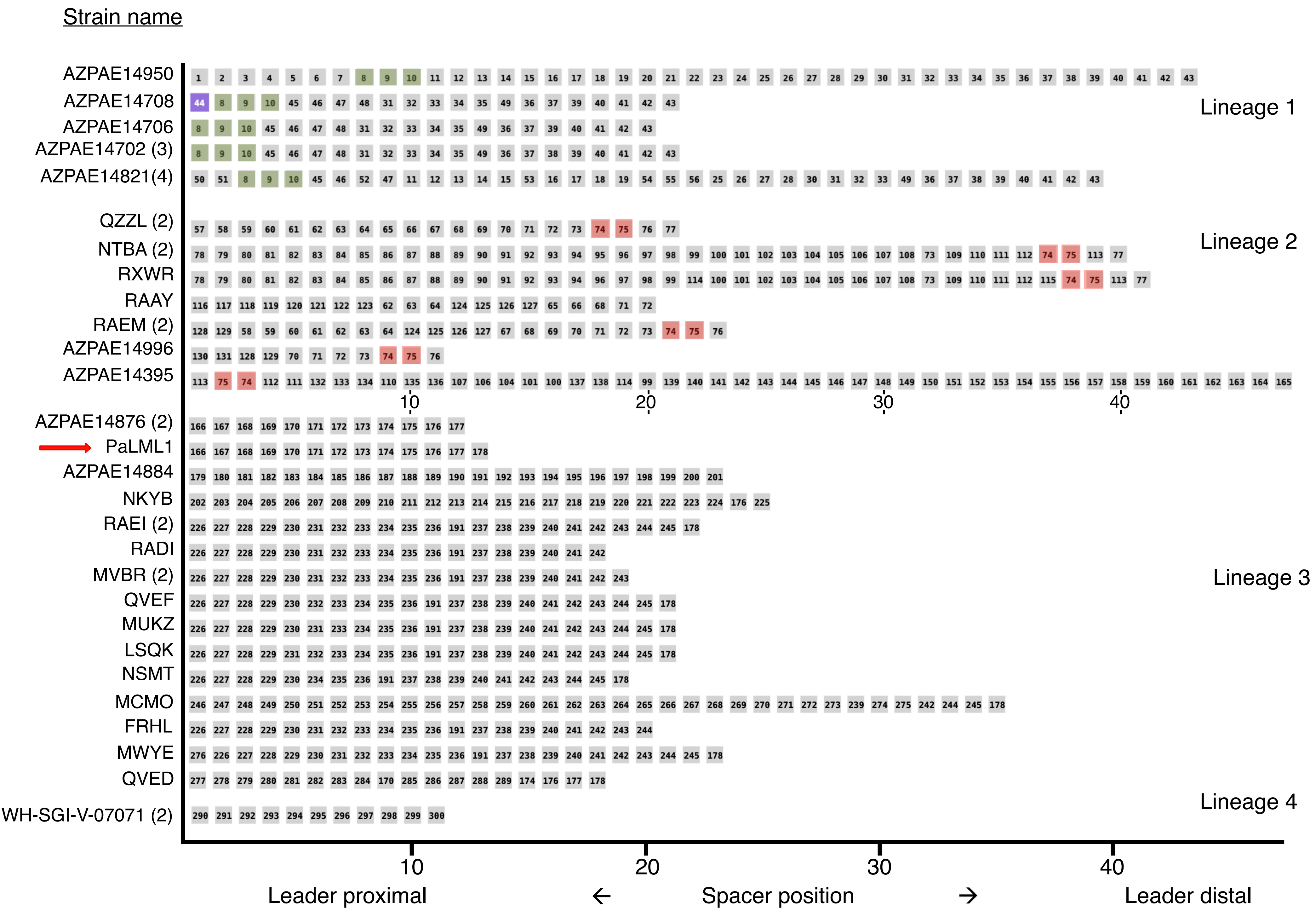

Supplemental Figure 1 **a.** Full CRISPR array lineage mapping of the 28 unique CRISPR arrays from 42 genomes. Each lineage contains CRISPR arrays that share *at least* one spacer. Spacers with the same DNA sequence are given the same number. Spacer #44 is a self-targeting spacer and is colored in purple.

Supplemental figure 2

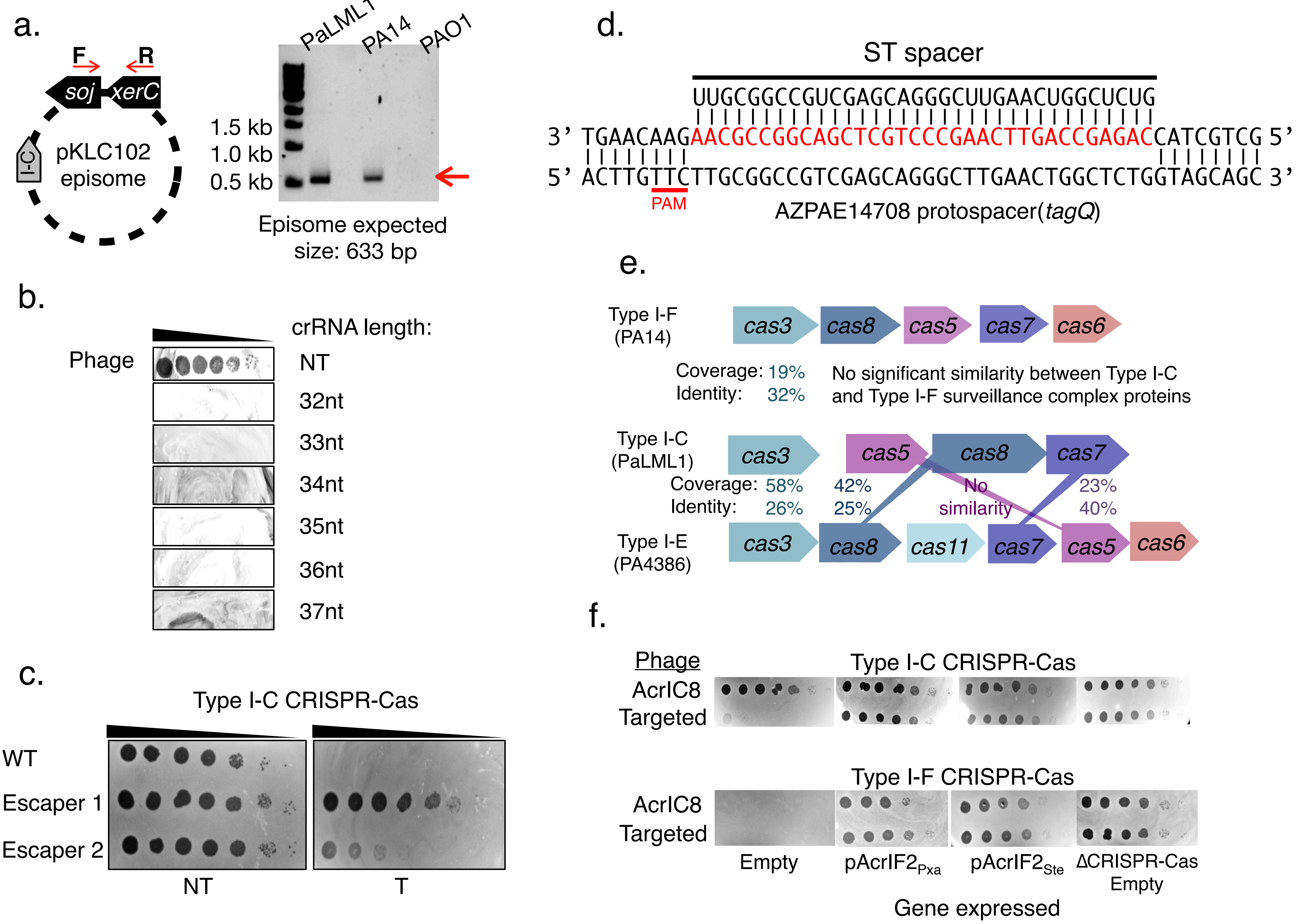

Supplemental Figure 2 **a.** The pKLC102 island can excise to form an episome. Red arrows represent forward and reverse primer pair used to analyze island excision. Gel depicts PCR products using the forward and reverse primer pair from schematic demonstrating the episomal nature of the pKLC102 element in PaLML1 and PA14 isolates. PAO1 does not harbor pKLC102 elements, resulting in no PCR product. **b.** Spot titration plaque assay of CRISPR-Cas sensitive phage serially diluted on a lawn of PaLML1, expressing crRNAs of lengths between 32-37 nt. The targeted phage is DMS3m, which does not have an acrIC gene. **c.** Spot titration plaque assay of WT (i.e. non escaper) phage and escaper phages 1 and 2 challenged with the Type I-C system in PAO1^{IC}. T = Targeting, NT = Non-targeting. Phages were spotted in 10x serial dilutions. **d.** Alignment of self-targeting spacer #1 from AZPAE14708 with corresponding protospacer. PAM is underlined in red. **e.** Comparison of Type I-F and Type I-E Cas protein sequences to Type I-C Cas protein sequences for the systems used in this study. Amino acid sequences can be found in Supplemental file 1.

f. Plaque assay testing the activities of two AcrIF2 homologues identified in *Pseudoxanthomonas* and *Stenotrophomonas* genomes. Homologues were expressed from a plasmid in either a strain encoding the Type I-C system (PAO1^{IC}, induced with 1mM IPTG) or the Type I-F system (PA14). A phage encoding a Type I-C Acr (AcrIC8) was used as a positive control, and a phage encoding AcrIIA4 (a Cas9 inhibitor) was used as the targeted phage.

Supplemental figure 3

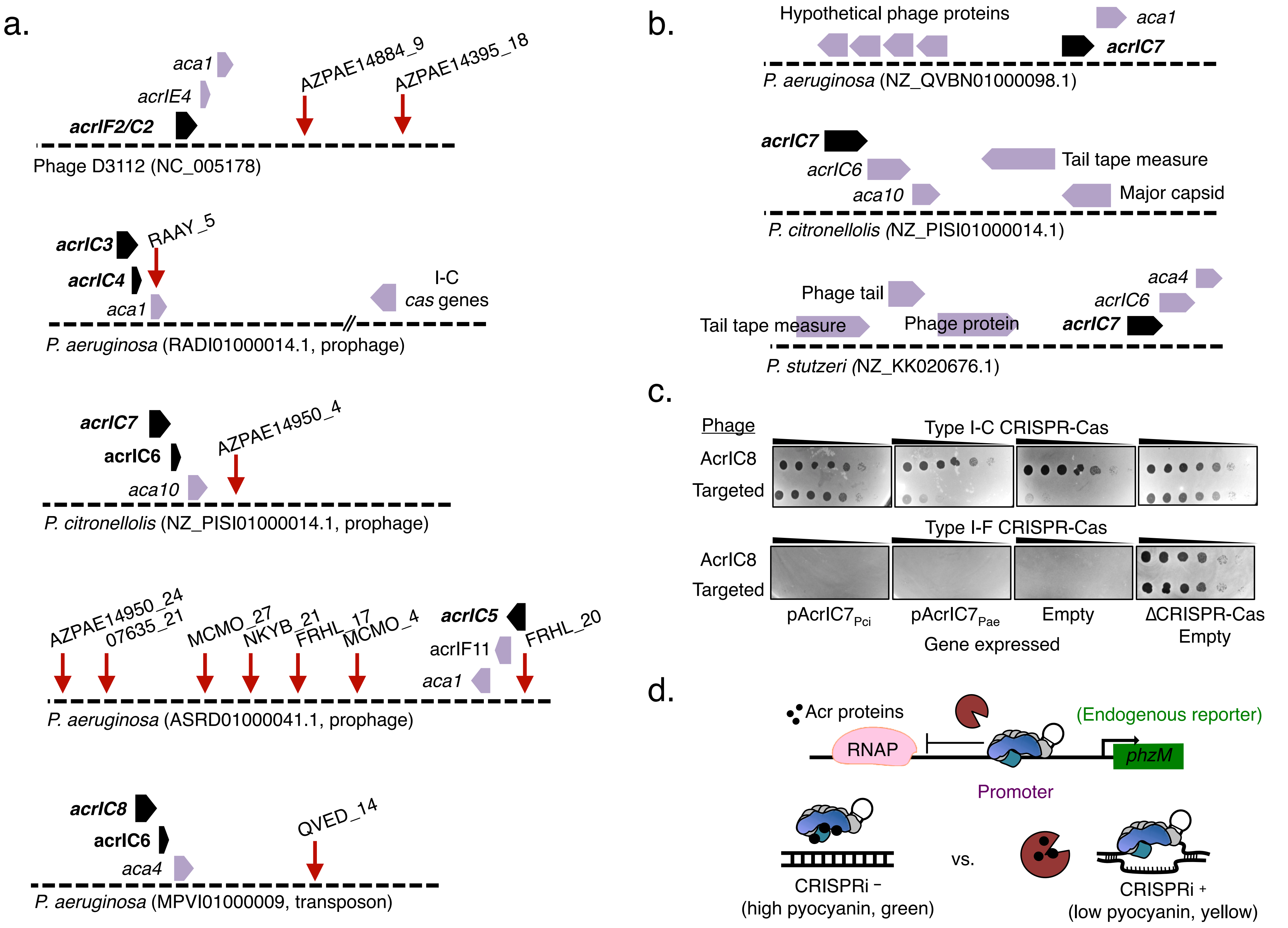

Supplemental Figure 3.

a. Schematic representation of the MGEs targeted by spacers found encoded by the Type I-C CRISPR arrays. Each MGE is shown with the Type I-C acr it encodes (black arrow) and relevant upstream and downstream genes. Spacer matches are indicated by a red arrow and the strain name plus spacer number. Accession number for the MGE is found below the genome annotation.

b. Loci showing typical genetic context of *acrIC7* in three *Pseudomonas* species. Genome accession code in parentheses.

c. Plaque assays of two AcrIC7 homologues expressed from a plasmid in $PAO1^{IC}$ or PA14. Acr activity was assessed by spotting a CRISPR-Cas sensitive phage in 10x serial dilutions (DMS3m expressing AcrIIA4) and an untargeted control (DMS3m expressing AcrIC8).

d. Schematic of the CRISPRi assay used to screen Acr activity. A crRNA is designed to bind upstream of *phzM*, a gene whose expression results in green pigmented *P. aeruginosa* cultures. Acrs that inhibit the surveillance complex from binding target DNA result in a CRISPRi- phenotype. Acrs that bind Cas3 or do not block DNA binding result in a CRISPRi+ phenotype.

Supplemental figure 4

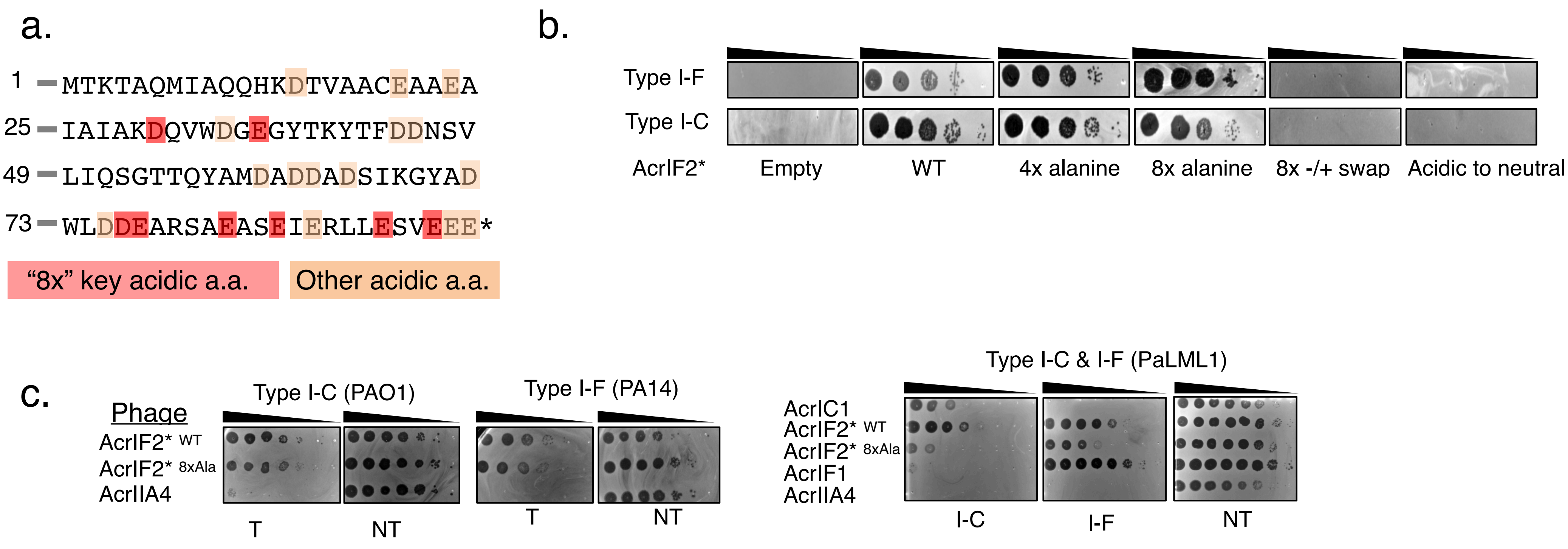

Supplemental Figure 4 **a.** AcrIF2* amino acid sequence shown with 8 key acidic residues (red) and all other acidic residues shaded (orange). These residues were mutated to either alanine, corresponding opposite charge residues, or corresponding neutral residues as indicated in the text. **b.** Plaque assays testing the activity of AcrIF2* mutants expressed from a plasmid. A I-F strain (PA14) or IC strain ($PAO1^{IC}$) were transformed with plasmids encoding the mutants indicated under each panel. Ten-fold serial dilutions of a CRISPR-Cas sensitive phage (DMS3m-AcrIIA4) was used to determine the activity of the AcrIF2* mutants. 4x alanine is D76A, D77A, E91A, and E94A. 8x mutant includes all mutated residues from the 4x mutant plus D30A, E36A, E82A, AND E85A. **c.** Plaque assays with ten-fold dilutions of the engineered mutant AcrIF2* phage tested in $PAO1^{IC}$ (Type I-C alone), PA14 (PA14 alone), or PaLML1 (both Type I-C and Type I-F co-expressed, with phage-specific crRNA provided on a plasmid and indicated below the appropriate panel). T= Targeting crRNA, NT= Non-targeting.

Supplemental figure 5

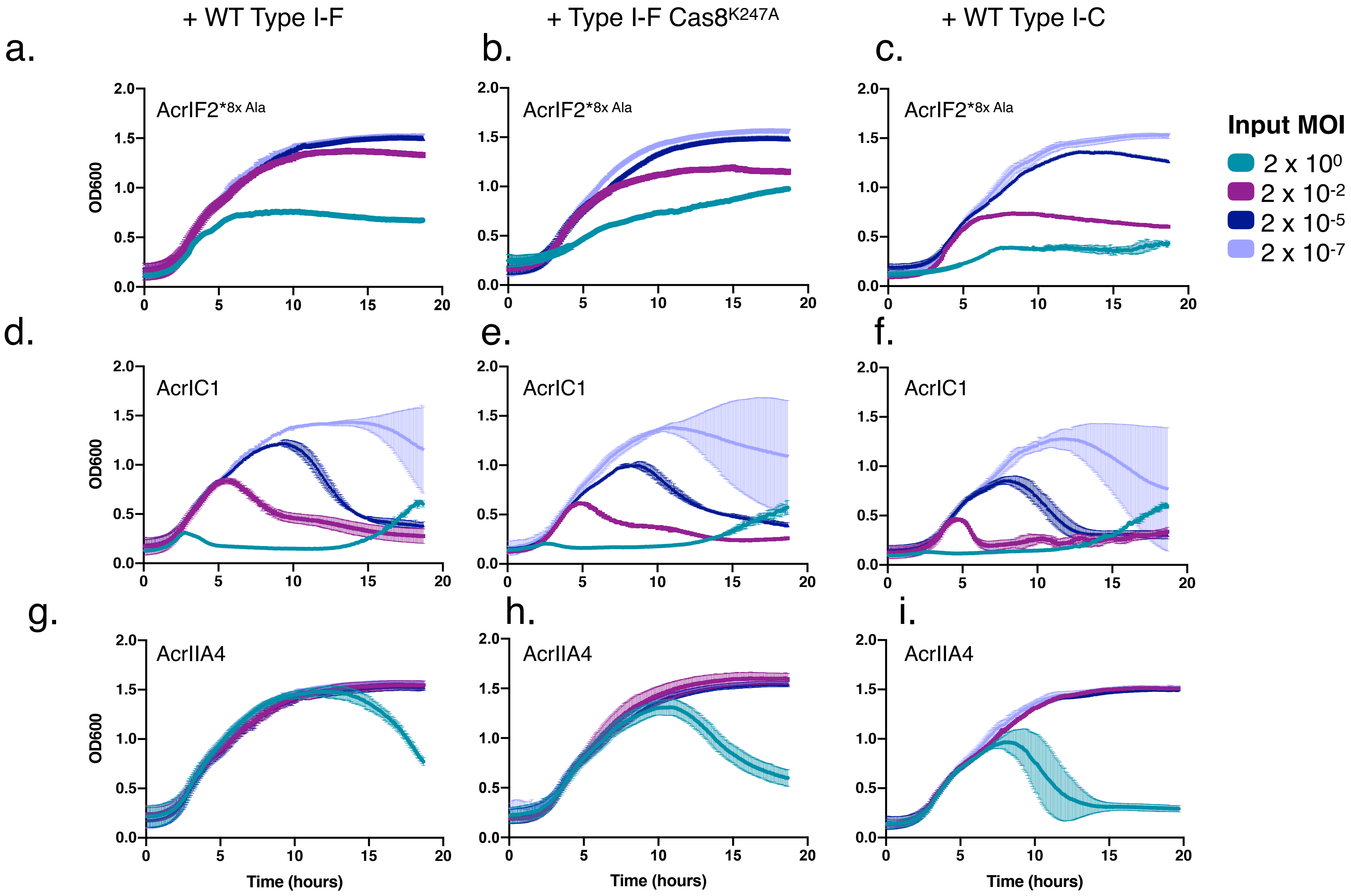

Supplemental Figure 5 **a-i.** Liquid infection assay with PAO1^{IC} transformed with indicated "decoy" surveillance complex plasmids (WT Type I-F, WT Type I-C, or Type I-F Cas8^{K247A}) and infected with a virulent DMS3m phage expressing AcrIF2*$^{8x\ Ala}$, AcrIC1, or AcrIIA4. Input phage MOI shown in legend.

Supplemental figure 6

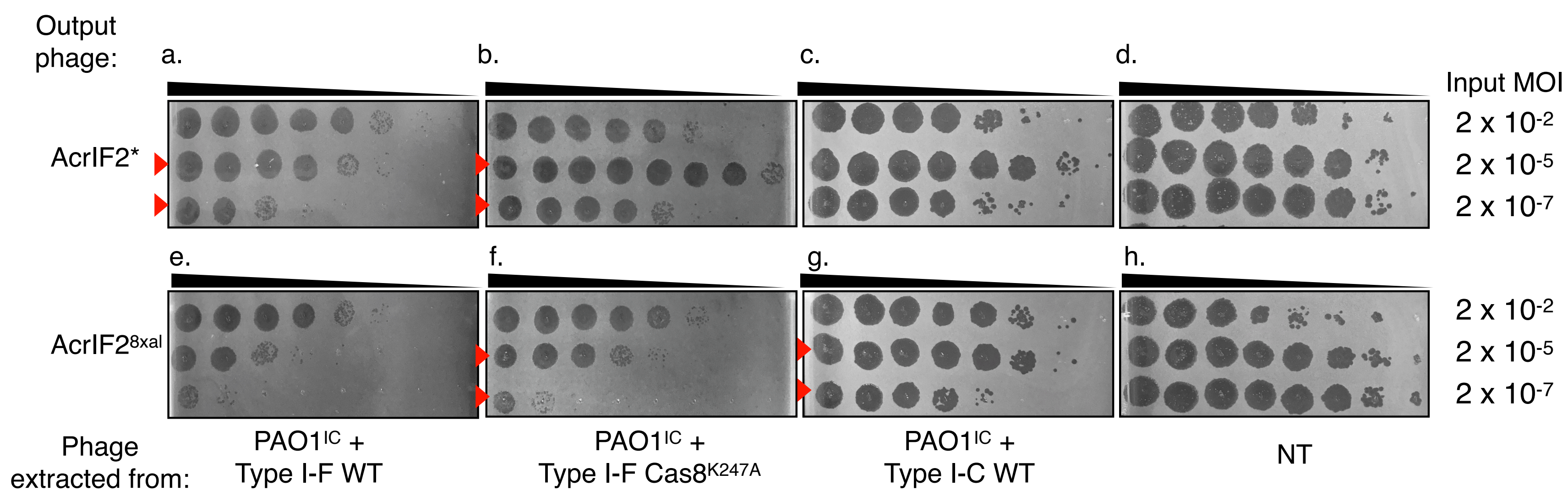

Supplemental Figure 6 **a-h.** Output phage following liquid infection assay from supplemental figure 5. Phage from cultures infected with virulent DMS3m expressing either AcrIF2* ^{WT} or AcrIF2* $^{8x\ Ala}$ was collected. 10x serial dilutions were spotted on an indicator strain (PAO1 WT). Input phage MOI shown in legend.

Supplemental figure 7

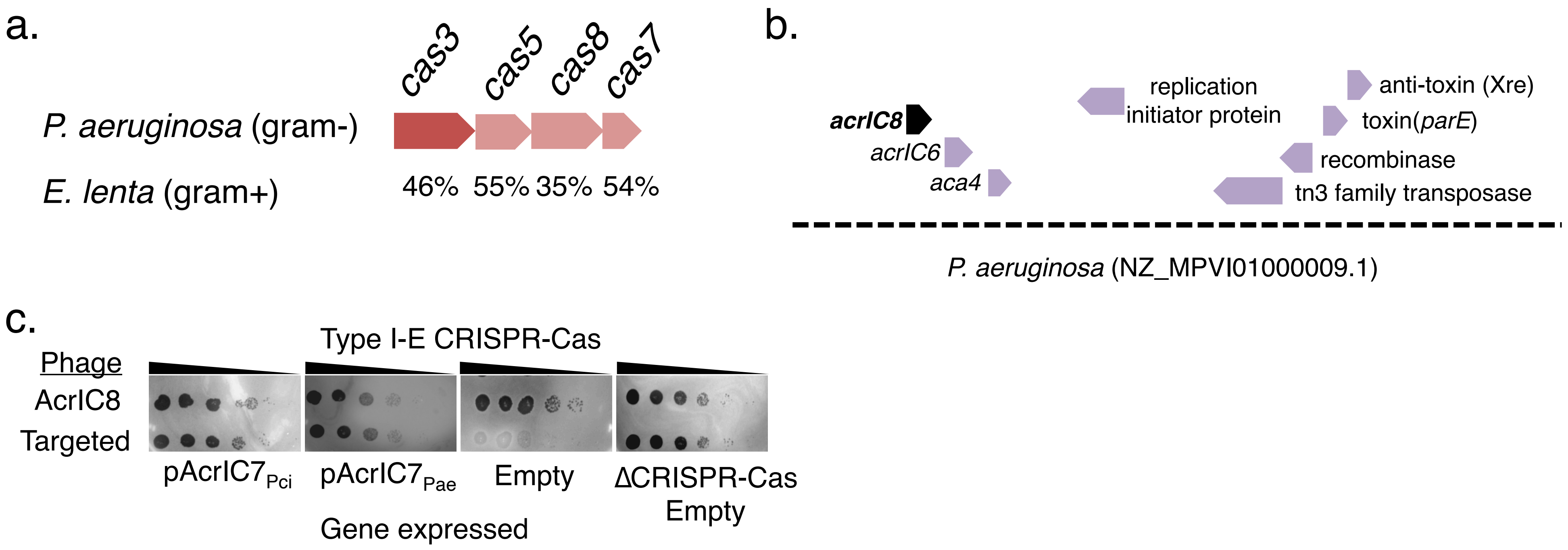

Supplemental Figure 7. **a.** Protein percent identity comparison of the *E. lenta* Type I-C CRISPR-Cas system to the *P. aeruginosa* Type I-C CRISPR-Cas system. **b.** Loci showing typical genetic context of *acrIC8*. **c.** Plaque assays of two AcrIC7 homologues expressed from a plasmid in PA4386. Acr activity was assessed by spotting a CRISPR-Cas sensitive phage in 10x serial dilutions (DMS3m expressing AcrIIA4) and an untargeted control (DMS3m expressing AcrIC8).
